# Rare case of upper gastrointestinal hemorrhage due to accessory spleen: A case report

**DOI:** 10.1097/MD.0000000000029636

**Published:** 2022-08-05

**Authors:** Yuanjun Liu, Yi Dai, Fan Xiao, Shuang Liu, Yakun Wu, Enrong Ran

**Affiliations:** a Department of Hepatobiliary Surgery, Suining Central Hospital, Suining, Sichuan Province, China; b Department of Nephrology, Suining Central Hospital, Suining, Sichuan Province, China.

**Keywords:** accessory spleen, case report, severe anemia, upper gastrointestinal hemorrhage

## Abstract

**Rationale:**

Upper gastrointestinal hemorrhage (UGIH) is defined as hemorrhage originating from the gastrointestinal tract proximal to the ligament of Treitz. The causes of UGIH include esophagitis, gastritis, peptic ulcers, Mallory–Weiss syndrome, and cancer. However, a rare cause of UGIH, such as an accessory spleen, may lead to serious complications if left untreated and can sometimes be very difficult to diagnose preoperatively.

**Patient concerns:**

An 18-year-old man was admitted to the Department of Gastroenterology of our hospital due to “repeated black stool for 2 months with aggravation, accompanied by hematemesis for 9 days.” He denied any history of hepatitis, trauma, or surgery.

**Diagnosis:**

Laboratory evaluation revealed severe anemia (hemoglobin, 6.4 g/dL). Computed tomography revealed a mass measuring 127 mm in its largest dimension, located in the upper left abdomen, with varicose veins in the gastric fundus. Moreover, distended blue–purple tortuous veins were observed by gastroscopy in the gastric fundus. We believed the mass was likely an abnormally proliferating accessory spleen; however, the causes of severe anemia and gastrointestinal hemorrhage were unknown.

**Interventions:**

After discussion in a multidisciplinary conference, the mass was completely resected laparoscopically, and the subserosal veins in the gastric fundus were sutured using absorbable threads.

**Outcomes:**

After the surgery, the patient recovered uneventfully without any complications. Clinicopathological examination showed that the mass was chronic congestive splenomegaly. Gastrointestinal hemorrhage secondary to an abnormally proliferating accessory spleen was confirmed as the diagnosis. Laboratory evaluation revealed hemoglobin at 12.1 g/dL 2 months after surgery. At the 12-month follow-up, the patient showed no recurrence of gastrointestinal hemorrhage.

**Lessons:**

UGIH caused by accessory spleen is extremely rare. This entity should be considered in differential diagnosis of gastrointestinal hemorrhage. Surgical intervention is necessary for timely diagnosis and treatment in case of gastrointestinal hemorrhage in critical clinical situations.

## 1. Introduction

Upper gastrointestinal hemorrhage (UGIH) is defined as hemorrhage originating from the esophagus, stomach, or duodenum.^[1]^ Common risk factors for UGIH include high-dose nonsteroidal anti-inflammatory drug use,^[2]^ anticoagulant use, and old age. The causes of UGIH include esophagitis, gastritis, peptic ulcers, Mallory–Weiss syndrome, and cancer.^[3]^ However, a rare cause of UGIH, such as an accessory spleen, may lead to serious complications if left untreated and can sometimes be very difficult to diagnose preoperatively. The spleen, which is fundamental for the hematological and immune system, is derived from mesenchymal cells of the dorsal mesentery.^[4]^ At the fifth week of embryonic development, an accessory spleen may form if the embryo spleen bud is not fully fused or if a single cell is separated from the body of the spleen.^[5]^ An accessory spleen is a congenital defect,^[6]^ 1 to 3 pieces, about 1.0 cm in diameter, normally located in the left upper abdomen, around the normal spleen. Abnormally enlarged accessory spleen may be found after splenectomy with or without hypersplenism. Preoperative diagnosis for an accessory spleen, whose location could vary, remains challenging, especially in emergencies.^[7]^ Here, we present a rare case of UGIH caused by an abnormally proliferating accessory spleen without trauma or splenectomy in medical history.

## 2. Case report

**Figure 1.**
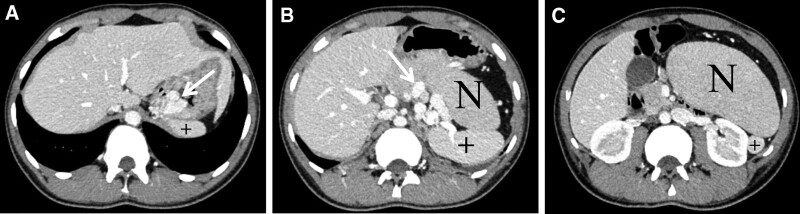
Dynamic contrast-enhanced CT scan findings (A–C). Solid arrow (→), varicose veins in the gastric fundus; asterisk (*), mass in the upper left abdomen; plus (+), normal spleen. CT = computed tomography.

**Figure 2.**
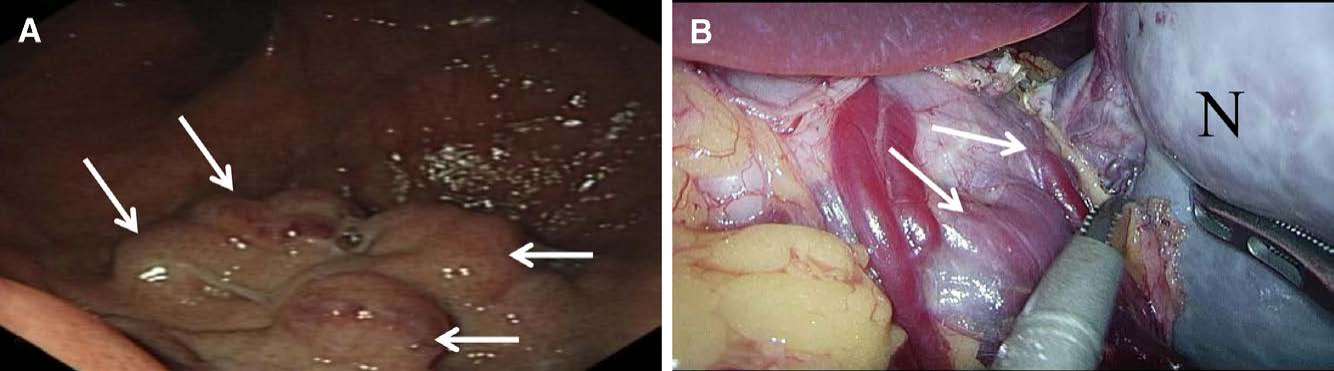
Gastroscopic findings (A). Solid arrow (→), distended blue–purple tortuous veins in the gastric fundus. Surgical findings (B). Solid arrow (→), subserosal blood vessels in the gastric fundus; asterisk (*), mass in the upper left abdomen.

An 18-year-old man was admitted to the Department of Gastroenterology of our hospital due to “repeated black stool for 2 months with aggravation, accompanied by hematemesis for 9 days.” The patient presented melena 2 months ago without obvious inducement, and the specific amount was unknown. No abdominal pain, acid reflux, abdominal distension, dizziness, fatigue, chills, fever, and other symptoms were noted. Nine days before admission, hematemesis occurred for unknown reasons. The volume was small, about 10 mL, and there was little blood in the saliva. One day before admission, the patient had unexplained hematemesis twice with a large amount of about 500 mL, vomiting bleeding clot, and defecated blood 3 times with a volume of about 800 g, accompanied by symptoms of dizziness and fatigue. No acid reflux, abdominal pain, abdominal distension, consciousness disorder, chills, fever, cough, expectoration, urgent urination, odynuria, and other symptoms were observed. Laboratory evaluation revealed severe anemia (hemoglobin, 6.4 g/dL). Computed tomography showed a mass measuring 127 mm in its largest dimension, located in the upper left abdomen, with varicose veins in the gastric fundus (Fig. 1). Additionally, distended blue–purple tortuous veins were observed by gastroscopy in the gastric fundus (Fig. 2A). Three years prior to this study, the patient was hospitalized in a large medical center for severe anemia, and bone marrow aspiration revealed active hyperplasia of the bone marrow. A genetic test for thalassemia did not exhibit any corresponding gene mutations. No abdominal mass was found 3 years ago. Anemia has been treated with oral “folic acid tablets, ferrous sulfate.” He denied histories of hepatitis, trauma, surgery, and food or drug allergy. There were no similar patients and no genetic predisposition in his family. After discussion in a multidisciplinary conference, the patient was considered for mass resection under laparoscopy. We believed the mass was likely an abnormally proliferating accessory spleen; however, the causes of severe anemia and gastrointestinal hemorrhage were unknown.

**Figure 3.**
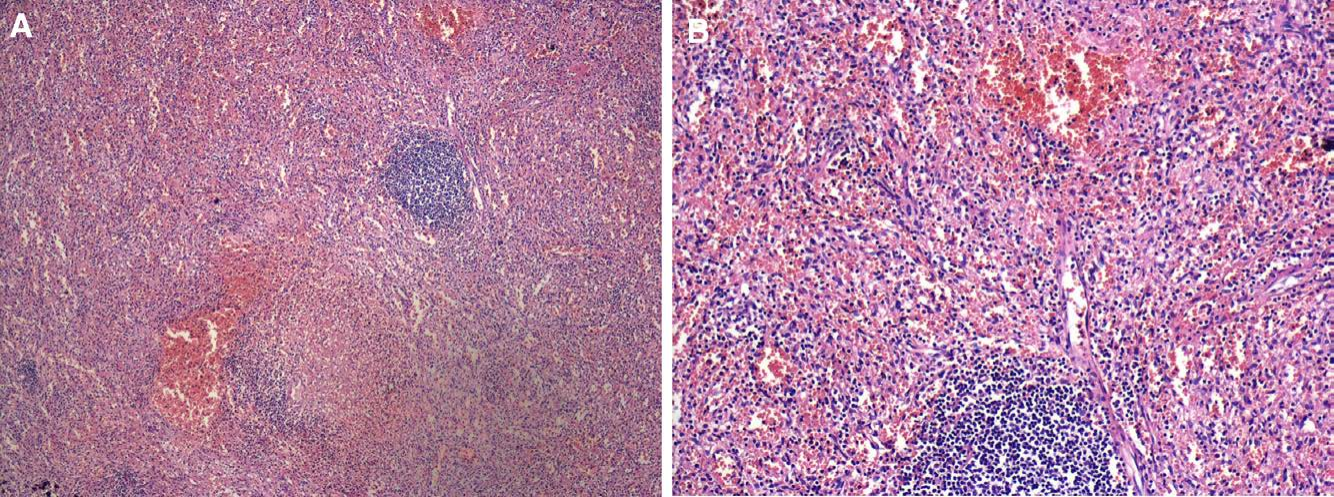
Clinicopathological examination showed chronic congestive splenomegaly. H&E × 40 (A). H&E × 100 (B). H&E = hematoxylin and eosin.

The mass was completely resected laparoscopically, and the subserosal veins in the gastric fundus were sutured using absorbable threads. During surgery, we found that the mass was closely related to the gastric fundus but separated from the pancreas, mesentery, and bowels. The distended tortuous veins of the mass were connected to the gastric fundus. The liver was soft, with no manifestation of cirrhosis and no ascites in the abdominal cavity (Fig. 2B). The small intestine, large intestine, and pancreas showed no obvious abnormalities, and the spleen was normal. After the surgery, the patient recovered uneventfully without any complications. Clinicopathological examination showed that the mass in the upper left abdomen corresponded to chronic congestive splenomegaly (Fig. 3). Gastrointestinal hemorrhage secondary to an abnormally proliferating accessory spleen was confirmed as the diagnosis. Laboratory evaluation revealed hemoglobin at 12.1 g/dL 2 months after surgery. At the 12-month follow-up, the patient showed no recurrence of gastrointestinal hemorrhage.

## 3. Discussion

An accessory spleen includes isolated splenic tissue outside the normal spleen,^[8]^ usually located at the tail of the pancreas and the splenic hilum but occasionally in the greater omentum and gastrointestinal tract.^[9]^ The location of the splenic tissue is often inconsistent with that of the congenital accessory spleen, and multiple sites may be involved.^[10]^ In contrast with a congenital accessory spleen, ectopic spleen implantation refers to the autologous transplantation of splenic tissue after splenectomy following trauma or other causes. This occurs in 67% of patients with splenic rupture and splenectomy.^[11]^ An accessory spleen is clinically crucial in some locations. When an accessory spleen is situated in another site, it may mimic some tumors, such as pancreatic and adrenal tumors.^[12]^ An accessory spleen is relatively common; however, it is rarely the cause of UGIH and severe anemia. In some cases, gastric splenosis may cause upper gastrointestinal bleeding, which is challenging to diagnose and is only incidentally found under most circumstances.^[13]^ While postoperative histopathologic examination is still the gold standard, fine magnetic resonance imaging and enhanced computed tomography scans may be useful in accessory spleen diagnosis.^[14]^

In the present case, the accessory spleen proliferated spontaneously in the presence of the normal spleen, and the cause was unknown. This was likely the cause of severe anemia in the patient 3 years prior to the study, or anemia was the cause of splenomegaly.^[15]^ It was also considered that the abnormally proliferating accessory spleen compressed the branches of the splenic vein, which could have caused the dilation and rupture of veins in the gastric fundus. After the accessory spleen enlarged further, its demand for blood supply increased, and, at the same time, the blood vessels entering and leaving the accessory spleen became enlarged. Because the blood supply of the abnormally proliferating accessory spleen came from the arteries of the greater curvature of the gastric body, its veins also directly returned to the submucosal vascular network of the greater curvature and the gastric fundus. Venous blood flow increased with the progressive enlargement of the accessory spleen, leading to the tortuous expansion of local blood vessels under the gastric fundus mucosa. The venous bulbs in the gastric fundus eventually ruptured, leading to UGIH. This possibility should be considered in differential diagnosis of gastrointestinal hemorrhage. Surgical intervention is necessary for timely diagnosis and treatment in the case of gastrointestinal hemorrhage in critical clinical situations.

Usually, an accessory spleen is not uncommon in clinical practice and does not cause serious problems. However, the abnormally proliferating accessory spleen in this case caused severe anemia to the point of shock symptoms. This case shows that the causes of severe anemia and gastrointestinal hemorrhage are diverse and complicated. The abnormally proliferating accessory spleen may be a possible cause, and clinical attention is warranted to distinguish it.

## Author contributions

**Conceptualization:** Yuanjun Liu, Enrong Ran.

**Investigation:** Yi Dai, Fan Xiao, Shuang Liu.

**Supervision:** Yakun Wu, Enrong Ran.

**Writing—original draft**: Yuanjun Liu.

**Writing—review & editing**: Yuanjun Liu, Yi Dai, Fan Xiao, Shuang Liu, Yakun Wu, Enrong Ran.

## Acknowledgments

We would like to thank the Department of Pathology, Suining Central Hospital for providing pathological images.
